# Providing lifestyle advice to women with PCOS: an overview of practical issues affecting success

**DOI:** 10.1186/s12902-021-00890-8

**Published:** 2021-11-23

**Authors:** Carolyn Ee, Stephanie Pirotta, Aya Mousa, Lisa Moran, Siew Lim

**Affiliations:** 1 NICM Health Research Institute, Western Sydney University, Locked Bag 1797, NSW 2751 Penrith, Australia; 2 Health and Social Care Unit, School of Public Health and Preventive Medicine, Monash University, 553 St. Kilda Road, VIC 3004 Melbourne, Australia; 3 Monash Centre for Health Research and Implementation - MCHRI, School of Public Health and Preventive Medicine, Monash University, Level 1, 43-51 Kanooka Grove, VIC 3168 Clayton, Australia

**Keywords:** Polycystic ovary syndrome, Lifestyle, Behaviour change, Obesity, Weight, Health literacy, Risk perception, Depression, Self-management, Fatigue, COM-B

## Abstract

Polycystic Ovary Syndrome (PCOS) is a common endocrine disorder affecting up to 13 % of women. Lifestyle interventions are first-line treatments, however attrition in women with PCOS is high. This review summarises current evidence on barriers to lifestyle management in PCOS and suggested strategies for overcoming these challenges, mapped to the Capability, Opportunity, Motivation and Behaviour model.

Physical capability for lifestyle changes may be impacted by altered gut hormone regulation and energy expenditure in PCOS. This may contribute to difficulties with weight management. The higher prevalence of eating disorders, disordered eating, fatigue and sleep disturbances are further barriers. Psychological capability may be reduced due psychological symptoms and lack of critical health literacy. Women with PCOS face similar challenges in terms of Opportunity to make lifestyle changes as other women of reproductive age. However, these are complicated by features more common in PCOS including body dissatisfaction. Motivation to adopt healthy lifestyles may be impacted by suboptimal risk perception and intrinsic motivation.

To address these barriers, screening for and management of eating disorders, disordered eating, depression, and Obstructive Sleep Apnoea should be undertaken as per international evidence-based guidelines. A weight-neutral approach may be appropriate with disordered eating. Building capability among health professionals to better partner with women with PCOS on their management is essential in addressing health literacy gaps. Behavioural strategies that target risk perception and build intrinsic motivation should be utilised. More research is required to understand optimal self-management strategies, risk perception, energy homeostasis and overcoming attrition in women with PCOS.

## Background

Polycystic Ovary Syndrome (PCOS) is a multisystem disorder with reproductive, metabolic, and psychological manifestations [1] affecting up to 13 % of women of reproductive age [2]. A woman is diagnosed with PCOS if she has any two of the three criteria established by the European Society for Human Reproduction and Embryology/American Society for Reproductive Medicine (ESHRE/ASRM) international consensus working group (also known as the Rotterdam criteria). These criteria are: hyperandrogenism (biochemical or clinical); irregular anovulatory cycles (>35 days or <21 days); and polycystic ovarian morphology [3]. Other reproductive disorders such as hyperprolactinemia must be excluded.

In addition to the reproductive features, PCOS is also associated with psychological and metabolic complications including decreased health-related quality of life [4] and increased prevalence of anxiety and depression [5], as well increased risk of future type 2 diabetes and coronary artery disease [6]. Insulin resistance is a key pathophysiological feature of PCOS and is present in even lean women with PCOS in a form that is proposed to be mechanistically distinct from obesity-associated insulin resistance [7]. Women with PCOS have a higher prevalence of overweight and obesity and a higher rate of longitudinal weight gain compared to women without PCOS [8]. Weight gain and obesity further worsen insulin resistance and consequently the overall features of PCOS [9]. Hence, weight management (weight gain prevention, modest weight loss or weight maintenance) is a first-line strategy as recommended in international evidence-based guidelines [10]. Such weight management strategies should encompass multidisciplinary lifestyle interventions, comprising of dietary, exercise and behavioural therapies [10].

While effective lifestyle modification is a key component of PCOS management, the evidence of higher obesity prevalence and longitudinal weight gain in community-recruited populations [11], combined with high attrition rates in clinical lifestyle interventions [12] suggests that women with PCOS experience challenges with weight management and implementing and sustaining lifestyle changes. Literature shows all women of reproductive age with or without PCOS are at greater risk of dropout when compared to women of older age [13]. This is likely due to factors such as family responsibilities, fatigue, lack of time, poor partner support, low mood and lack of exercise facilities to name a few. The overlap in barriers in all women of reproductive age suggests attrition is due to life-stage rather than a PCOS diagnosis [14]. In this paper, we present a review of barriers to lifestyle management in women with PCOS and suggested strategies to overcome these challenges. Briefly, we searched the MEDLINE database from inception until March 2021 using the following keywords: Polycystic Ovary Syndrome OR PCOS, appetite, energy expenditure, peptide yy, ghrelin, glucagon like peptide 1, basal metabolic rate, post prandial thermogenesis, respiratory quotient, health literacy, management AND lifestyle, implementation AND lifestyle, facilitators, barriers, qualitative, disordered eating, eating disorders, self-management, risk perception, perceived risk, fatigue, sleep, obstructive sleep apnoea OR OSA, anxiety, depression, body image distress, psychological. We mapped these barriers and strategies against the Capability, Opportunity, Motivation and Behaviour (COMB) model, which posits that one’s ability to engage in a behaviour (*capability)*, the enabling circumstance (*opportunity)*, and the willingness to engage (*motivation)* all need to be present for a particular behaviour to occur [15]. Developed based on 19 behaviour change frameworks, the COMB model consists of a hub (*Capability*, *opportunity* and *motivation/COM*) and a ‘Behaviour Change Wheel’, around which nine intervention functions are positioned, which inform strategies to behaviour change corresponding to the COM domains [16].

## Barriers in capability, opportunity, motivation and behaviour

### Capability: physical

#### Appetite regulation and energy expenditure

There are a number of potential physiological reasons unique to women with PCOS that may explain difficulties with weight management. Insulin resistance and hyperinsulinaemia may predispose women with PCOS to gain weight, supported by insulin therapy-inducing weight gain [17] through anabolic effects of insulin and changes in energy expenditure, glycosuria, and food intake [18–23]. Androgen excess may favour abdominal fat deposition [24], which could additionally contribute to insulin resistance in PCOS.

There may also be abnormalities in energy homeostasis in women with PCOS. The limited literature in this area has reported decreases in basal metabolic rate [25] or postprandial thermogenesis [26] for women with PCOS compared to those without, although this is not consistently found [26–29]. The respiratory exchange ratio or respiratory quotient (RQ) refers to substrate oxidation or the carbohydrate-to-fat oxidation ratio [30], with a higher RQ (indicative of reduced fat oxidation) previously associated with weight gain [31, 32] in the general population. Women with PCOS have been reported to have higher RQ [33] compared to women without PCOS. Metabolic flexibility refers to the metabolic capacity to switch from lipid oxidation in fasting conditions to lipid availability in non-fasting conditions and is assessed by comparing RQ in the fasting state and an insulin rich state. A systematic review of five studies reported higher metabolic inflexibility in women with PCOS compared to controls and similar metabolic inflexibility compared to women with type 2 diabetes or obesity [34]. Further, these alterations in energy expenditure or metabolic inflexibility have been associated with insulin resistance [25, 26, 34] and hyperandrogenism [34].

Appetite regulation has also been reported to be impaired in women with PCOS, with alterations in hunger and gut hormones. A systematic review of 20 studies involving 894 women with and 574 women without PCOS reported lower ghrelin in PCOS (standardised mean difference -0.40; 95 % confidence interval -0.73, -0.08) [35], related to factors including insulin resistance. There is evidence that suggests intra-adipose androgen excess plays a role in the cycle of insulin resistance and weight gain [36]. Postprandial cholecystokinin has also been reported to be reduced [37] and fasting or postprandial glucagon-like peptide-1 (GLP-1) increased [38] or decreased [39] in women with PCOS. Further, women with PCOS have been reported to be less satiated and more hungry after eating compared to women without PCOS [40] and have a higher energy intake and earlier return of hunger [41]. However, the literature again is inconsistent, with other studies reporting no differences in gut hormones or appetite in women with and without PCOS [38, 42–44]. Of note, there is also research reporting that there may be additional mechanisms contributing to increased adiposity in PCOS, such as altered regulation of appetite regulatory brain regions occurring in conjunction with insulin resistance [45]. There is therefore an emerging, albeit inconsistent, body of research suggesting mechanisms for why obesity and weight gain may be increased in this population.

#### Eating disorders

Women with PCOS are reported to have a higher prevalence of both disordered eating behaviours [46–48] and eating disorders [49, 50] compared to women without PCOS in the majority of, but not all, prior literature [51–54]. In the limited research to date, studies use a variety of screening tools [e.g. Eating Attitudes Test-26 [47], the Mini International Neuropsychiatric Interview (MINI) [55] and the Eating Disorder Examination Questionnaire (EDE-Q) [56], with each having a different screening criterion. This lack of uniformity in assessments contributes to the reason as to why further clarification is required to better understand whether disordered eating or eating disorders are more prevalent in women with PCOS compared to those without the condition. Furthermore there is a lack of understanding as to why women with PCOS may experience greater rates of disordered eating behaviours and eating disorders compared to women without PCOS. A recent Australian study explored the cross-sectional relationship between disordered eating or eating disorders and a range of demographic risk factors (PCOS diagnosis, body mass index [BMI], age, country of birth). Authors reported that disordered eating, but not eating disorders, were more prevalent in women with PCOS when compared with controls [57]. Of the disorders, binge-eating disorder (BED) was the most commonly reported but not significantly different to controls. BED is the most common form of eating disorders within the general population and may explain the frequency and lack of significance between the two groups [58]. The cross-sectional study also reported higher BMI and older age increased the odds of disordered eating whilst increased BMI and younger age increased the odds of an eating disorder [57]. It is important to highlight that the controls were psychology university students and may not be a true representation of the general population. A meta-analysis also reported that women with PCOS are at 3.05 greater odds of being diagnosed with an eating disorder compared with women without PCOS, but was unable to distinguish the specific subtypes of eating disorders diagnosed due to a lack in power. More rigorous studies investigating the prevalence and presentation of disordered eating and eating disorders in women with PCOS are needed to better inform screening and management.

#### Fatigue and sleep

Fatigue has been reported by women with PCOS as a barrier to adopting lifestyle changes [14], which may be due to physiological or psychological origins. Women with PCOS are more likely to report fatigue than women without PCOS [59]. Fatigue is intrinsically linked with sleep, and women with PCOS have a higher prevalence of poor sleep quality and sleep disorders compared to women without [60–62]. In particular, it is estimated that up to 35 % of women with PCOS may have obstructive sleep apnoea (OSA) [62], compared to 9-38 % in the general population [63]. Women who are obese and of older age are more at risk of developing OSA [62]. Sleep disturbances in women with PCOS may also occur independently of OSA [64, 65] and may be related to psychological distress and upregulation of the hypothalamic-pituitary axis, and/or increased sympathetic outflow due to insulin resistance [66] although exact mechanisms are still unclear.

### Capability: psychological

#### Depression and depressive symptoms

Fatigue and sleep disturbances may also indicate depression, and are the most commonly reported depressive symptoms in women with PCOS [59]. The prevalence of depression and depressive symptoms is higher in PCOS [67] and PCOS is also associated with more severe symptoms of depression [68]. A recent meta-analysis of 57 studies reporting on 172,040 women reported that women with PCOS were at 2.79 greater odds for being clinically diagnosed with depression when compared to controls [68]. Another meta-analysis estimated a prevalence of 36 % for depression in PCOS [69]. The risk of developing depression may be associated with higher BMI [70], although this association is weak. The development of depression and depressive symptoms may also be related to the presence of hyperandrogenism, hyperinsulinemia and elevated levels of inflammation in PCOS [71].

#### Body image distress

Women with PCOS are more likely to report a lack of confidence about maintaining physical activity [72] which may be related to negative body image, and feelings of shame and worthlessness [73]. While body image distress is not limited to women with PCOS [74], there is a high prevalence of body image distress among women with PCOS and it has been suggested that this might mediate the development of depression and anxiety [75]. Research suggests that women with PCOS experience feelings of being inferior and abnormal compared with women without PCOS [76] and this may lead to beliefs that they are unable to participate in physical activity [73]. All of these factors may contribute to difficulties initiating and maintaining lifestyle changes.

#### Self-management

Self-management is a continuous process of self-directed behaviour change known to improve one’s personal emotional, behavioural and medical management, aiming to avoid disease-related complications, regulate symptoms and reduce the severity of disease [77–79]. These actions may take form as cognitive (e.g. problem solving, decision making) and/or behavioural strategies (e.g. forming relationships with health practitioners and having healthy foods easily available) to meet one’s own health needs [79, 80]. All of these core self-management behaviours are heavily influenced by one’s knowledge [81] and self-efficacy [82] and must be targeted in order to prevent or manage chronic disease. Effective self-management strategies in the general population include the independent monitoring of symptoms, development of personalised action plans upon worsening of symptoms, understanding and implementing psychological coping strategies to better manage stress, and enhancing personal responsibility in daily lifestyle choices and medication adherence [83]. Unfortunately, the use of self-management strategies and their impact on nutrition and physical activity are yet to be investigated in women with PCOS.

#### Health literacy

Health literacy provides a comprehensive lens on self-management among women with PCOS. Health literacy is an important determinant of health equity and comprises a multi-dimensional concept described by the World Health Organisation as “the cognitive and social skills which determine the motivation and ability of individuals to gain access to, understand and use information in ways which promote and maintain good health” [84]. Research in the general population found that health literacy is associated with health behaviours and health outcomes including hospitalisation rates, overall health status, obesity, dietary intake, and physical activity [85–88]. As PCOS is a complex disorder with variable and wide-ranging symptoms, its self-management is likely to also be complex requiring high levels functional, interactive, and critical health literacy. However, there has been very limited research on health literacy in women with PCOS, as discussed below [89, 90].

There are three main categories of health literacy: functional, interactive and critical health literacy [91]. While evidence presented here are on the individual types of health literacy, dynamic interactions exist between the different types of health literacies. For example, poor knowledge on PCOS (functional health literacy) could limit meaningful engagement with health professionals (interactive health literacy); on the other hand, poor engagement of allied health professionals (interactive health literacy) limit opportunities to obtain the appropriate knowledge and skills in lifestyle management (functional health literacy).

##### Functional health literacy in PCOS

*Functional* health literacy describes the basic knowledge and skills in health including literacy and numeracy skills to process health information such as reading food labels [92]. Higher levels of functional health literacy were associated with lower BMI in women with PCOS as shown in one study in women from Arabic backgrounds [89]. Past studies have also shown that women with PCOS attempt a wide range of weight loss approaches on their own [93–95]. This results in an unstructured approach usually lacking in key behaviour change techniques such as goal setting and self-monitoring such as keeping a food diary [90, 96]. The omission of key behaviour change strategies could explain the lack of weight loss success frequently reported by women with PCOS [90].

##### Interactive health literacy in PCOS

*Interactive* health literacy describes the ability to apply the knowledge through supportive relationships, which could include accessing health services and support, the ability to engage healthcare professionals to manage health and having social support for health [91, 97]. In terms of their interaction with health professionals, women with PCOS often report challenges and frustration around delayed diagnosis of PCOS, inadequate and inconsistent information and poor communication between healthcare professionals and women with PCOS [98–100]. This suggests poor interactive health literacy in the self-management of women with PCOS. Peer support is another important aspect of interactive health literacy, comprising an important part of supportive relationships in the management of PCOS. There is increasing recognition of the role of PCOS support groups in promoting awareness, advocacy and general support among its members [101]. However, women with PCOS have reported a sense of isolation and lack of access to support from peers [102].

##### Critical health literacy in PCOS

*Critical* health literacy builds on functional and interactive health literacies to advocate for individual and social health, such as making shared decisions on one’s health with health professionals [91, 103, 104]. In our previous study on health literacy in women with PCOS, none of the participants attributed their past successes in lifestyle and weight management to a strong partnership with a healthcare professional [90], which is an essential component of critical health literacy [91, 103, 104]. As this health literacy category builds on capacities drawn from functional and interactive health literacies, significant gaps in the latter, particularly those around relationships with healthcare providers, may have prevented the development of a partnership with healthcare professionals. Similar research and practice gaps in the domain of critical health literacy have been highlighted in lifestyle management of other women of reproductive age due to a lack of patient involvement in intervention development [105].

### Opportunity

Women with PCOS report similar barriers to lifestyle and weight management as those generally faced by women of reproductive age without PCOS, including conflict with work and family commitments, or disliking the “mundaneness” of a restricted diet [14, 106]. These barriers may contribute to the high risk of weight gain in women of reproductive age [107]. However, these general barriers are further complicated with features of PCOS, particularly the psychological manifestations of body dissatisfaction and depressive symptoms. For example, not having a suitable place to exercise intersects with feeling embarrassed about being seen outdoors or in a gym, and this in turn is related to negative thoughts about feeling depressed [108]. Thus, reduced opportunity to exercise due to the lack of safe environment to do so is further compounded by psychological issues and self-perception in PCOS.

### Motivation

#### Risk perception

Risk perception is one’s subjective judgement about the likelihood that an adverse event will occur and the severity of consequences should it occur [109]. Risk perceptions are a precursor to health-related behaviours as they influence how one views disease and the protective actions they may or may not take to reduce risk [110, 111]. There is limited understanding regarding the health-related knowledge and beliefs of women with PCOS. Despite poorer overall health and greater health-related impediments in women with PCOS, the few studies published to date report conflicting findings regarding the health-related beliefs of women with PCOS compared with those without [112, 113]. For instance, some studies report that risk perceptions of future metabolic complications such as diabetes and cardiovascular disease (CVD) are suboptimal in women with PCOS [113], while others do not [114, 115]. A recent study of 475 women [116] reported that, compared with healthy controls, women with PCOS had a greater perceived risk for adverse health outcomes and weight gain, yet believed a healthy lifestyle was less beneficial for preventing weight gain.

Notwithstanding their own perceptions of risk, evidence suggests that PCOS is associated with greater risk of metabolic diseases such as type 2 diabetes in the long term [117, 118]. Moreover, despite their unique risk profiles, women with PCOS are often provided with general population government dietary recommendations that they perceive as inadequate and dismissive of their PCOS needs [115, 119, 120]. As a result, women with PCOS tend to be ambivalent about meeting these dietary recommendations, with only a few following them, even though they reported high confidence in being able to adopt the recommendations and being more concerned about developing adverse health outcomes compared to women without PCOS [116].

#### Intrinsic motivation

Women with PCOS also appear to lack intrinsic motivation to undertake lifestyle change, such as enjoyment of physical activity, instead viewing physical activity only as a means to weight loss. This is evidenced by previous research reporting that a major barrier to physical activity adoption among women with PCOS is the perceived barrier that exercise is tiring and hard work [121], and fear of injury [72].

## Behaviour: attrition

All the barriers in *Capability, Opportunity and Motivation* contribute to the key behavioural issue underlying lifestyle management in PCOS—attrition. Attrition is a common barrier to successful weight management, reported across all weight management interventions targeting women of reproductive age with or without PCOS [122]. Women with PCOS may experience additional challenges, with attrition as high as 46 % [123] in this population, compared to 30 % in clinical weight loss interventions in women without PCOS [12, 124–126].

It is known that individuals who prematurely terminate weight loss interventions do not attain the required skills and strategies to effectively overcome barriers to lifestyle change. This impacts long term weight management, with intervention completion positively correlated with weight loss [127]. Other health implications include a reduced quality of life (physical, mental, and social wellbeing), increased loss of income due to work absenteeism [128–130], and an increased risk of obesity-associated disease [127, 131].

To date, literature has identified participant characteristics (e.g. younger age, lower BMI, male, unemployed status and lower education) [132] and predictors (e.g. increased previous weight loss attempts, poorer quality of life, more stringent weight outcomes, lower carbohydrate intake, increased stress, and lower state of motivation to change) [122, 132, 133] that may lead to attrition in the general population [134]. However, no research has been conducted on the differential predictors of attrition in women with or without PCOS. Only one study reported that depression was associated with attrition in PCOS [124].

## Implications for research and practice in capability, opportunity and motivation

### Capability: physical

#### Appetite regulation and energy expenditure

When communicating to women with PCOS, it is important to be aware of these mechanisms as to best acknowledge the potential pathophysiology of challenges they may experience when trying to manage weight. Further research is needed both in women with PCOS and the general population on strategies to aid weight management through modulating energy expenditure or appetite regulation.

#### Fatigue and sleep disturbance

If fatigue, sleepiness and non-restorative sleep are identified, screening for OSA should be considered, followed by a referral to a sleep specialist if required. The tool recommended by international evidence-based guidelines on PCOS is the Berlin Questionnaire [135], although this has not been validated in PCOS. Fatigue and sleep disturbances may also be due to depression, and screening for depression should be conducted as required, per evidence-based guidelines (see Table 1). Fatigue and sleep disturbances may also be due to depression, and screening for depression should be conducted as required, per evidence-based guidelines (see Table 1). Women with sleep disturbances that are not due to OSA may still benefit from a sleep specialist referral, and sleep hygiene should be prescribed. Cognitive behavioural therapy may also be helpful [66].

**Table 1:** Recommended screening for depression, negative body image and eating disorders [136]

Assessment of anxiety and or depressive symptoms involves assessment of risk factors, symptoms and severity. Symptoms can be screened according to regional guidelines, or by using the following stepped approach: **Step 1:** Initial questions could include: Over the last 2 weeks, how often have you been bothered by the following problems? • feeling down, depressed, or hopeless? • little interest or pleasure in doing things? • feeling nervous, anxious or on edge? • not being able to stop or control worrying? **Criteria:** If any of the responses are positive, further screening should involve: ● assessment of risk factors and symptoms using age, culturally and regionally appropriate tools, such as the Patient Health Questionnaire (PHQ) or the Generalised Anxiety Disorder Scale (GAD7) and/or refer to an appropriate professional for further assessment.	
Negative body image, can be screened according to regional guidelines or by using the following stepped approach: **Step 1:** Initial questions could include: • Do you worry a lot about the way you look and wish you could think about it less? • On a typical day, do you spend more than 1 hour per day worrying about your appearance? (More than 1 hour a day is considered excessive) • What specific concerns do you have about your appearance? • What effect does it have on your life? • Does it make it hard to do your work or be with your friends and family? **Criteria:** If an issue is identified, health professionals could further assess by: • Identifying any focus of concern of the patient and respond appropriately • Assessing the level of depression and/or anxiety • Identifying distortion of body image or disordered eating	
Eating disorders and disordered eating can be screened using the following stepped approach. **Step 1:** The SCOFF (Sick, Control, One stone, Fat, Food) screening tool can be used or initial screening questions can include: ◦ Does your weight affect the way you feel about yourself? ◦ Are you satisfied with your eating patterns? **Criteria:** If the SCOFF tool or any of these questions are positive, further screening should involve: ◦ assessment of risk factors and symptoms using age, culturally and regionally appropriate tools; ◦ referral to an appropriate health professional for further mental health assessment and diagnostic interview. If this is not the patient’s usual healthcare provider, inform the primary care physician.	

#### Eating disorders and disordered eating

Lifestyle recommendations may need to be sensitive to disordered eating or eating disorders so as to not perpetuate disordered eating psychopathologies [48]. Eating disorders are psychological disorders characterised by abnormal or disturbed eating behaviours with or without compensatory behaviours, as determined by the Diagnostic and Statistical Manual of Mental Disorders-5 [137]. Behaviours that classify an eating disorder may include food restriction, binge-eating, purging, laxative use, diet pills and excessive exercise. Disordered eating is a condition characterised by these same characteristics but are of a lesser frequency or a lower level of severity as that of an eating disorder [137]. One example in which lifestyle recommendations may be applied whilst not exacerbating disordered eating symptomologies is by incorporating a weight-neutral approach (e.g. intuitive eating, self-care activities) [57, 138], rather than the traditional, dietary restriction. A 2019 systematic review found weight-neutral programs reduced serum lipid measures and blood pressure comparable to weight loss interventions. In both weight-neutral and weight loss focus programs non-significant reductions in HbA1c were observed [139]. Further studies are needed to confirm the impact of weight-neutral programs in women with PCOS. Furthermore, understanding the demographic risk factors associated with these pathological eating behaviours may help clinicians identify women with PCOS most at risk of eating disorders or disordered eating. Until more research is available, clinicians are recommended to screen all women with PCOS for possible disordered eating behaviours, with particular attention to women with elevated BMI (see Table 1). Moreover, future research should incorporate interviews with a clinical psychologist to clinically diagnose disordered eating and eating disorders.

### Capability: psychological

#### Self-management

As lifestyle interventions and weight management are key recommendations for the management of PCOS, identifying the most effective and acceptable lifestyle-related self-management strategies in this population may help support them in achieving lifestyle change and meeting the nutrition and physical activity recommendations as outlined in the 2018 evidence-based clinical guidelines for optimal PCOS management [140]. This will also inform the tailoring of PCOS lifestyle interventions for subgroups such as by BMI, age or medical history.

#### Depression and body image distress

Evidence-based guidelines recommend that all women with PCOS should be routinely screened for anxiety and depression symptoms at diagnosis and referred for psychological therapy if required. Initial screening questions and follow-up screening are provided in Table 1. Negative body image, which has been associated with poor sleep, can also be screened using a stepped approach, outlined in Table 1. Approaches to reduce body image distress include counselling based on acceptance and commitment therapy [141]. As with disordered eating and eating disorders, a weight-neutral approach is recommended for women with psychological co-morbidities.

#### Health literacy

Involving women with PCOS in the co-development of health interventions or decisions is key to addressing the gap in critical health literacy, as demonstrated in recent research [142]. Better partnership between health professionals and women with PCOS has been highlighted as one of the key tenets of PCOS management in the latest evidence-based guidelines [10]. Building capacity among health professionals to be better partners with women with PCOS is among the strategic activities in the translation of the guidelines.

Wider adoption of evidence-based tool such as the AskPCOS smartphone application could improve communication between women with PCOS and health professionals (thereby improving interactive health literacy), since it includes a question prompt list that enables women with PCOS to discuss targeted health concerns with their healthcare providers [143, 144]. Additionally, further strategies to support peer groups should be developed and sustained to improve interactive health literacy in women with PCOS.

### Opportunity

Considering the barriers of lack of time, childcare responsibilities and work commitment in women of reproductive age [14, 106], intervention targeting this group need to be flexible in structure and delivery to ensure engagement. Previous studies have seen success in low-intensity lifestyle intervention for weight gain prevention in women with young children [145], however, higher intensity interventions may be needed for weight loss [146]. The involvement of peers in exchanging ideas on how to overcome barriers to create more opportunities for healthy eating and physical activity within the context of the lives of women with PCOS may also be helpful [101] to expand opportunity, including using persuasion and incentivisation. Incentivisation may take the form of financial benefits, such as weekly financial incentives, return of initial deposits according to the number of sessions attended or completion of a program or, group incentives based on the attendance of individuals as a collective in each program group [147].

### Motivation

#### Risk perception and other barriers

Despite limited evidence, there is a clear need for increased education, awareness, and accuracy of risk perception among women with PCOS. Shared decision-making and an improved understanding of the features of risk (including individual responses to risk aversion, risk immediacy, perceived vulnerabilities, consequences and control) are important considerations for successfully implementing lifestyle change [148]. However, adequate risk perception is insufficient on its own, and must be coupled with appropriate tools and resources to enable individuals to take preventive action [109]. Focusing on the reported successes of weight loss interventions in women with PCOS and providing strong encouragement about health-related issues may represent important targets for health professionals when counselling women with PCOS about the benefits of lifestyle modification. Further effort is required to communicate risks of unhealthy lifestyles to women with PCOS. Consistent with this approach, lifestyle messages for women with PCOS should be tailored to reflect the unique risk profiles of PCOS in terms of cardiometabolic disease prevention.

#### Building intrinsic motivation

Women should be encouraged to choose physical activities that they find enjoyable, rather than the ones they think they are expected to engage in (such as going to the gym). The use of mindfulness-based therapies is a potentially novel intervention that may help to enhance intrinsic motivation to exercise, and may have additional benefits for mental health [149].

## Behaviour

### Overcoming attrition

Our recent meta-analysis on strategies to reduce attrition in weight loss interventions for the general population included 57 studies and 7581 participants and reported that intervention strategies such as multi-component interventions (nutrition, physical activity and behavioural strategies combined), monetary incentives (e.g. return of deposit following program completion or financial reward), and self-monitoring significantly reduce the risk of attrition in weight loss interventions[147]. Multi-component interventions included the use of weekly group sessions and behavioural technique education, behaviour change counselling and program reminders, self-monitoring and anti-stress management, self-monitoring with feedback or a chat platform for peer and professional support, weekly payments with pre-packaged foods, weekly group dietitian and psychology sessions, and energy restriction coupled with self-monitoring and behavioural techniques. Self-monitoring encompassed paper and electronic devices to provide instant generalised or personalised feedback.

Within a clinical setting, financial incentives could be small cash payments, lottery tickets, grocery vouchers, or reduced-fee follow-up appointments to reward clinical attendance [147, 150–152]. Incentives greater than 1.2 % of disposable income may be most beneficial to reduce attrition [153]. Research from the King’s Fund in the United Kingdom found that the use of financial incentives are feasible in clinical settings but a mixture of positive (e.g. payment for attendance) and negative incentives (e.g. removal of refund or deposit return) must be included for optimal attendance and long-term behaviour change outcome[154].

Our systematic review highlighted a current lack of studies reporting attrition in female-only weight loss lifestyle interventions and we were unable to conduct subgroup analyses to determine the most effective attrition reducing strategies in females[147]. In another study we reported that women with PCOS presenting with depression symptoms upon program initiation are at greater odds of drop-out across lifestyle interventions [124]. Further studies aiming to assess the impact of attrition on behaviour change and weight loss in lifestyle interventions among females in both the general population and women with PCOS are needed [147]. Identifying intervention design factors that influence attrition in women with PCOS will help facilitate overall PCOS intervention effectiveness, helping to reduce the risk of PCOS associated disease [147].

## Conclusions

There are a number of barriers to successful lifestyle change among women with PCOS. Emerging research suggests that a range of intrinsic factors for weight management may be altered in PCOS affecting the capability of women with PCOS to manage their weight, although this evidence is limited and currently inconsistent. Opportunity may be reduced due to systemic and general barriers which are compounded by PCOS-specific factors such as negative body image. Altered risk perception and a lack of intrinsic motivation (i.e. enjoyment) may affect motivation in women with PCOS. In assisting women to achieve their lifestyle goals, a multidisciplinary and multicomponent approach is required, incorporating self-management, behaviour change techniques to overcome barriers, screening and referral as appropriate for depression, negative body image, and eating disorders as per evidence-based guidelines, and overcoming health literacy gaps. More research needs to be conducted on overcoming attrition, gaps in risk perception, use of financial incentives and on tailoring recommendations for lifestyle change to be specific to women with PCOS.

## Data Availability

Not applicable.

## Abbreviations

BED: Binge-eating disorder
BMI: Body mass index
COMB: Capability Opportunity Motivation Behaviour model
CVD: Cardiovascular disease
GLP-1: Glucagon-like peptide 1
OSA: Obstructive sleep apnoea
PCOS: Polycystic Ovary Syndrome
RQ: Respiratory quotient
